# Combined Scintigraphy and Tumor Marker Analysis Predicts Unfavorable Histopathology of Neuroblastic Tumors with High Accuracy

**DOI:** 10.1371/journal.pone.0132809

**Published:** 2015-07-15

**Authors:** Wolfgang Peter Fendler, Vera Wenter, Henriette Ingrid Thornton, Harun Ilhan, Dietrich von Schweinitz, Eva Coppenrath, Irene Schmid, Peter Bartenstein, Thomas Pfluger

**Affiliations:** 1 Department of Nuclear Medicine, Ludwig-Maximilians-University of Munich, Munich, Germany; 2 Department of Pediatric Surgery, Ludwig-Maximilians-University of Munich, Munich, Germany; 3 Institute of Clinical Radiology, Ludwig-Maximilians-University of Munich, Munich, Germany; 4 Department of Pediatric Haematology and Oncology, Ludwig-Maximilians-University of Munich, Munich, Germany; University of Pécs Medical School, HUNGARY

## Abstract

**Objectives:**

Our aim was to improve the prediction of unfavorable histopathology (UH) in neuroblastic tumors through combined imaging and biochemical parameters.

**Methods:**

^123^I-MIBG SPECT and MRI was performed before surgical resection or biopsy in 47 consecutive pediatric patients with neuroblastic tumor. Semi-quantitative tumor-to-liver count-rate ratio (TLCRR), MRI tumor size and margins, urine catecholamine and NSE blood levels of neuron specific enolase (NSE) were recorded. Accuracy of single and combined variables for prediction of UH was tested by ROC analysis with Bonferroni correction.

**Results:**

34 of 47 patients had UH based on the International Neuroblastoma Pathology Classification (INPC). TLCRR and serum NSE both predicted UH with moderate accuracy. Optimal cut-off for TLCRR was 2.0, resulting in 68% sensitivity and 100% specificity (AUC-ROC 0.86, p < 0.001). Optimal cut-off for NSE was 25.8 ng/ml, resulting in 74% sensitivity and 85% specificity (AUC-ROC 0.81, p = 0.001). Combination of TLCRR/NSE criteria reduced false negative findings from 11/9 to only five, with improved sensitivity and specificity of 85% (AUC-ROC 0.85, p < 0.001).

**Conclusion:**

Strong ^123^I-MIBG uptake and high serum level of NSE were each predictive of UH. Combined analysis of both parameters improved the prediction of UH in patients with neuroblastic tumor. MRI parameters and urine catecholamine levels did not predict UH.

## Introduction

Based on the grade of Schwannian stromal development, neuroblastic tumors are divided into three histopathologic categories: neuroblastoma, ganglioneuroblastoma and ganglioneuroma [1]. Risk of progression and mortality in patients with neuroblastic tumors is critically determined by their histopathology, but also by tumor genetics and the extent of disease at primary staging [2–3]. All three factors have been included in The International Neuroblastoma Risk Group (INRG) Classification System [4]. Risk stratification according to INRG is critical for therapeutic decision making, since patients with undifferentiated neuroblastoma require more aggressive treatment than do patients with, for example, ganglioneuroma [5]. Risk stratification of neuroblastic tumors is usually performed through histological examination of tumor specimens after surgery. Pre-surgical and non-invasive risk estimation by imaging or serum tumor markers may contribute important information with the potential to guide neo-adjuvant therapy. Magnetic resonance imaging (MRI), scintigraphy using meta-[^123^I]-iodobenzylguanidine (^123^I-MIBG), as well as blood and urine tests are routinely performed for pre-operative staging. Each of these examinations determines a different risk factor for prediction of tumor aggressiveness: In particular, we have recently linked findings for semiquantitative ^123^I-MIBG uptake on single-photon emission computed tomography (SPECT) to results from tumor histopathology [6]. Here we made a semiquantitative assessment of ^123^I-MIBG uptake as the tumor-to-liver ratio on SPECT images, an index which is less susceptible to misassignment of uptake from overlying liver or intestines, as can occur in planar scintigraphy images. Results of other trials over the past few decades have linked tumor marker concentrations in serum and urine to histopathology and patient outcome [7–11]. In the case of MRI, image-defined risk factors (IDRF) can indicate the probability of malignancy [12]. However, these three types of investigations have limited diagnostic accuracy for prediction of tumor phenotype. We hypothesized that a combined analysis of imaging and tumor marker characteristics might overcome this limitation, so as to improve substantially the overall diagnostic accuracy for prediction of high-risk histopathology in patients with neuroblastic tumor.

## Materials and Methods

### Patients

Patients were included retrospectively. Inclusion criteria were (a) histopathologically proven neuroblastic tumor; (b) scintigraphy, MRI and urine/blood tests before surgery/biopsy; (c) a maximum interval of 50 days between any diagnostic test (scintigraphy, MRI, or serum/urine tests) and surgery/biopsy; and (d) no tumor-directed treatment between diagnostic tests and surgery/biopsy. 47 consecutive pediatric patients with staging examinations between January 2006 and January 2012 met our criteria. The retrospective study protocol was approved by the local ethics committee (Ethikkommission der Medizinischen Fakultät der LMU München), and written informed consent for entry into the study was waived. Written informed consent (as outlined in PLOS consent form) was obtained from legal guardians of two individuals to publish case details. Mean time interval between scintigraphy and MRI/tumor marker/surgery or biopsy was 5/3/12 days (range: 0–50 days). Patients were staged according to the International Neuroblastoma Staging System (INSS) [2].

### Serum NSE and urine catecholamines

24 hour urine samples were collected in a container prefilled with hydrochloric acid (10%). Urine levels of creatinine, the catecholamines dopamine (DP), noradrenaline (NA), and adrenaline (A) and their metabolites metanephrine (MN), vanillylmandelic acid (VMA), and homovanillic acid (HVA) were measured by high-performance liquid chromatography (Chromsystems, Martinsried, Germany). Blood samples were drawn at the end of urine collection and serum levels of neuron-specific enolase (NSE) were determined by an automated Elecsys 2010 System (Roche Diagnostics, Mannheim, Germany).

### Imaging

Scintigraphy was performed under thyroid blockade with p.o. sodium perchlorate at a dose of 10 mg/kg/day over three days. The first sodium perchlorate dose was administered 30–60 min before application of ^123^I-MIBG, which was administered i.v. at a dose adapted to body weight according to specifications of the European Association of Nuclear Medicine (EANM) [13]. Images were acquired using a dual-head gamma camera Prism 2000 XP (Picker Inc, Cleveland, OH, USA) starting 24h after tracer administration. Spot images of the whole body dorsal/ventral view were obtained in a matrix of 256 x 256 pixels with a maximum acquisition time of ten minutes per image. SPECT images of the tumor-affected regions were acquired in all cases at approximately 24h after tracer administration (3° steps, 2 x 180°, 15 seconds per step). Iterative reconstruction of the SPECT was based on an OSEM algorithm (3 iterations, 15 subsets, 128 x 128 matrix).

MRI was performed on a 1.5 T scanner (Magnetom Vision, Siemens, Erlangen, Germany). The field-of-view encompassed the region suspected for tumor involvement. Axial, coronal, or sagittal short tau inversion recovery (STIR) images and spin echo or fast spin echo T2-weighted and T1-weighted images with and without administration of gadopentetate dimeglumine (Magnevist, Schering, Berlin, Germany; 0.2 ml/kg of body weight) were obtained. To avoid motion artifacts, light sedation was necessary in four of the 47 patients. Legal guardians of all patients gave standard written consent to undergo scintigraphy and MRI.

### Image analysis

Images were reviewed on a HERMES workstation (Nuclear Diagnostics, Haegersten, Sweden). Tumor lesions were identified on MRI images at the primary site as any mass adjacent to the adrenal glands, in a paravertebral location, or in other tumor-typical regions of the head, thorax, abdomen or pelvis. Margins of the mass were judged as either (a) well-defined, referring to a well circumscribed lesion or (b) ill-defined, referring to an irregularly shaped lesion with infiltration or encasement of adjacent structures. Images were fused using the HERMES software algorithm. Side-by-side and fused MRI images were used to localize lesions on SPECT. Largest MRI diameter and the tumor-to-liver count-rate ratio (TLCRR) on ^123^I-MIBG SPECT were determined for all patients, who had in part been reported in our previous study [6]. We modified our TLCRR metric in order to minimize the effects of signal noise and tissue inhomogeneity, but following closely in our new criteria the PERCIST 1.0 definition for tumor and liver uptake [14]: Peak tumor count rate was defined as the average count rate in a 3 cm^3^ spherical VOI around the voxel with highest count rate in the tumor, which gives a high tumor-related tracer activity concentration without excessive statistical noise. We elected to use a volume three times larger than the PERCIST definition to accommodate the lower resolution of SPECT versus PET. In accordance with PERCIST, we defined liver background count rate as the mean activity concentration in a 3 cm diameter spherical ROI placed in the central right hepatic lobe or, in case of hepatic metastases, in other non-diseased areas of the liver. TLCRR was defined as peak tumor count rate divided by the liver background. Image analyses were performed by two experienced readers (W.F. and T.P.). MRI and scintigraphy were reviewed separately by both readers with knowledge of clinical data, but without knowledge of the histopathological findings. In case of any disagreement, images were reviewed by both readers together and consensus decision was made after discussion of the case.

### Histopathology

Tumors were classified in accordance with the International Neuroblastoma Pathology Classification System (INPC) on the basis of the proportion of undifferentiated tumor cells into the following groups [15]: (1) Ganglioneuroma (Schwannian stroma-dominant); Ganglioneuroblastoma, of which either (2) intermixed (Schwannian stroma-rich) or (3) nodular (Schwannian stroma-rich/stroma dominant and stroma-poor); (4) Neuroblastoma (Schwannian stroma-poor). Based on INPC criteria, assignment to groups 1 or 2 was judged favorable, whereas groups 3 and 4 were judged as unfavorable [15]. Resected samples were examined separately at the Department of Pathology of the Ludwig-Maximilians-University of Munich, Germany and at the German Pediatric Tumor Registry of the Department of Pediatric Pathology, University of Kiel; the separate analysis did not in any individual patient result in discordant findings.

### Statistical analysis

Characteristics are presented as number (percent) or mean ± standard deviation (SD). Parameters had been tested for normality using the Shapiro-Wilk test, which rejected the null-hypothesis of normal distribution. Therefore, the Mann-Whitney test was used for unpaired comparison between two subgroups. Area under the receiver-operating-characteristic (AUC-ROC) curve, 95% confidence interval (CI) and corresponding p values were calculated for prediction of UH. No more than ten variables were included, ensuring a minimum ratio of about five individuals for every variable [16]. Significance was set at *p < 0.005 in accordance with Bonferroni correction for multiple tests (*p < 0.05 divided by ten, the number of variables). Optimal cut-off was defined as the point on the ROC curve which is farthest from the line of equality (Youden index). A bar chart was drawn in ascending order for individual values including the optimal cut-off value to illustrate the number of false positive and false negative findings for each significant variable. Sensitivity (SE), specificity (SP), positive predictive value (PPV), negative predictive value (NPV), accuracy (AC), and relative risk (RR) were calculated for significant variables using the optimal cut-off as determined by ROC analysis. The SPSS software package (version 15.0, SPSS, Inc., Chicago, Illinois, USA) was used for statistical analyses.

## Results

### Characteristics of the study cohort

Patient characteristics are given in Table 1. Median age was two years and six months (range: 1 month– 19 years and 9 months). 31 of 47 (66%) primary tumors were neuroblastoma and 41 of 47 (87%) were located within the abdomen. 33 of 47 patients (70%) had INSS neuroblastoma disease stage III and IV based on histopathology and imaging findings at diagnosis. Tumor marker concentration in patients with favorable (FH) versus unfavorable histopathology (UH) and corresponding p values are given in Table 2. According to the Mann-Whitney test with correction for multiple comparisons only serum NSE was significantly elevated in the UH group, whereas all other tumor markers showed no significant difference.

**Table 1:** Patient characteristics. INSS = International Neuroblastoma Staging System.

Patient characteristic (n = 47)		Absolute number (percent)
INPC group		
	Ganglioneuroma	9 (19)
	Ganglioneuroblastoma intermixed	4 (9)
	Ganglioneuroblastoma nodular	3 (6)
	Neuroblastoma	31 (66)
INSS stage		
	I	7 (15)
	IIA	3 (6)
	IIB	4 (9)
	III	11 (23)
	IV	18 (38)
	IVS	4 (9)

**Table 2:** Urine catecholamine metabolite levels and serum NSE in patients with favorable histopathology (FH) and unfavorable histopathology (UH) according to the International Neuroblastoma Pathology Classification (INPC). Concentrations are given as mean ± standard deviation (SD). Difference was tested by unpaired Mann-Whitney test including Bonferroni correction for multiple comparisons (p < 0.005*). MN = metanephrine, VMA = vanillylmandelic acid, A = adrenaline, NA = noradrenaline, HVA = homovanillic acid, DP = dopamine, NSE = neuron-specific enolase.

Tumor marker (n = 47)	FH	UH	p
MN (mg/g)	1.1 ± 1.1	3.3 ± 5.6	0.036
VMA (mg/g)	11.2 ± 6.5	54.4 ± 123.1	0.157
A (μg/g)	10.6 ± 8.9	12.8 ± 33.6	0.161
NA (μg/g)	69.1 ± 49.7	142.0 ± 335.9	0.686
HVA (mg/g)	15.1 ± 7.4	33.0 ± 43.4	0.433
DP (mg/g)	878.8 ± 576.8	1729.0 ± 3193.7	0.784
NSE (ng/ml)	23.0 ± 4.9	55.3 ± 74.0	0.001*

### ROC analysis of image parameters and tumor marker concentrations

**Fig 1:**
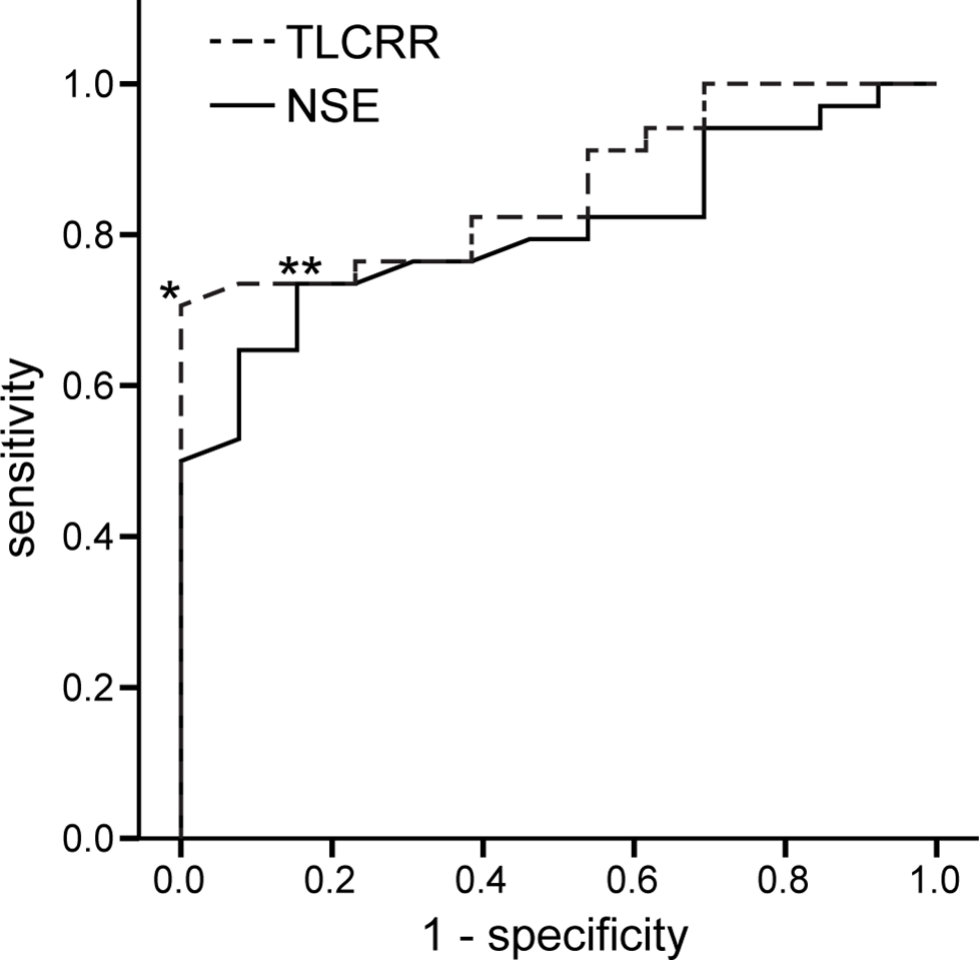
ROC of TLCRR and serum NSE for prediction of unfavorable histopathology. Receiver-operating-characteristic (ROC) is given for TLCRR (dashed line) and serum NSE (continuous line). AUC-ROC was 0.86 for TLCRR. Optimal cut-off determined by the Youden index was 2.0 (*), resulting in a sensitivity of 68% and a specificity of 100%. AUC-ROC was 0.81 for serum NSE. Optimal cut-off was 25.8 ng/ml (**) with a sensitivity of 74% and a specificity of 85%. AUC-ROC = area under the receiver-operating-characteristic, TLCRR = tumor-to-liver count-rate ratio, NSE = neuron-specific enolase.

**Fig 2:**
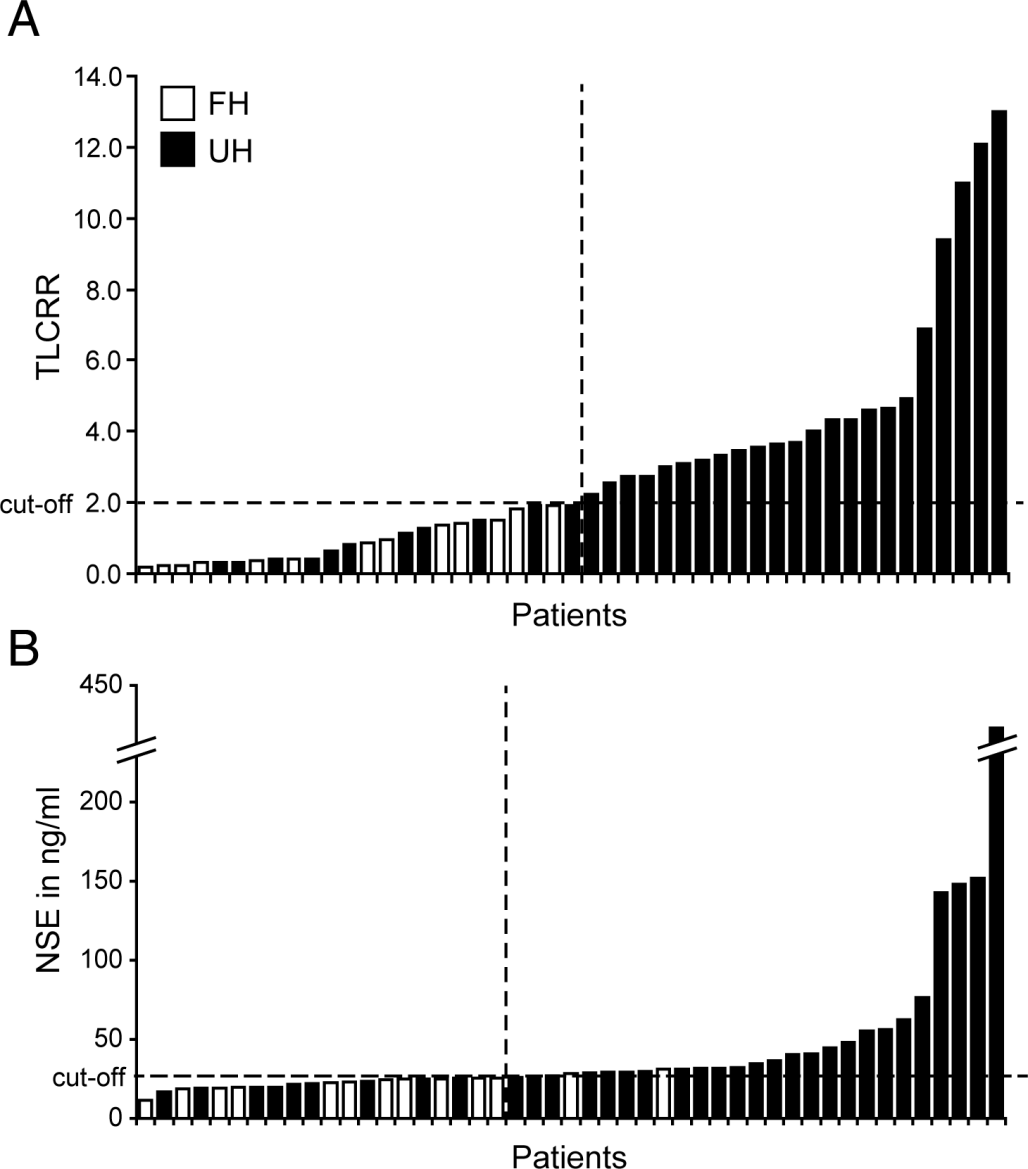
Results for (A) TLCRR and (B) serum NSE with corresponding histopathology for each patient (n = 47). Patients with unfavorable histopathology (UH) are shown with black bars, and patients with favorable histopathology (FH) are shown with white bars, all of which are ordered in ascending magnitude. Cut-off values determined by ROC analysis are indicated by horizontal lines. ROC = receiver-operating-characteristic, TLCRR = tumor-to-liver count-rate ratio, NSE = neuron-specific enolase.

A total of ten parameters were analyzed for their accuracy to predict UH. These were MRI size, MRI margins, ^123^I-MIBG TLCRR, serum NSE, and urine concentration of three catecholamines and three catecholamine metabolites, normalized by 24 hour creatinine levels. Results are given in Table 3. Type of MRI margins separated favorable and unfavorable histopathology with an AUC-ROC of 0.68, although accuracy of prediction did not reach statistical significance for this parameter. TLCRR, MN and serum NSE each had an AUC-ROC ≥ 0.70 with p < 0.05. However, only TLCRR and serum NSE were considered significant after Bonferroni correction for multiple tests (p < 0.005*; AUC-ROC ≥ 0.80). The Youden index revealed an optimal cut-off of 2.0 for TLCRR and 25.8 ng/ml for serum NSE. ROC curves and optimal cut-off for prediction of UH are illustrated in Fig 1. Bar charts in ascending order are shown in Fig 2 separately for TLCRR and serum NSE. False positive/false negative results were seen in 0/11 cases for TLCRR and in 2/9 cases for serum NSE.

**Table 3:** Results of the ROC analysis. Accuracy of several imaging parameters and tumor markers for prediction of unfavorable histopathology was tested by ROC analysis using Bonferroni correction for multiple comparisons. Corresponding AUC-ROC, p value and 95% CI are given.

			95% CI	
Parameter (n = 47)	AUC-ROC	p	upper limit	lower limit
MRI size	0.40	0.279	0.24	0.56
MRI margins	0.68	0.053	0.51	0.86
TLCRR	0.86**	< 0.001*	0.76	0.97
MN	0.70	0.036	0.53	0.87
VMA	0.63	0.157	0.48	0.79
A	0.37	0.161	0.19	0.55
NA	0.54	0.686	0.37	0.71
HVA	0.57	0.433	0.41	0.73
DP	0.47	0.784	0.31	0.64
NSE	0.81**	0.001*	0.68	0.93

**AUC-ROC > 0,80.

*p < 0.005 in accordance with Bonferroni correction.

ROC = receiver-operating-characteristic, AUC = area under the curve, CI = confidence interval, MRI = magnetic resonance imaging, TLCRR = tumor-to-liver count-rate ratio, MN = metanephrine, VMA = vanillylmandelic acid, A = adrenaline, NA = noradrenaline, HVA = homovanillic acid, DP = dopamine, NSE = neuron-specific enolase.

### Diagnostic accuracy of separate versus combined TLCRR and NSE analysis

**Fig 3:**
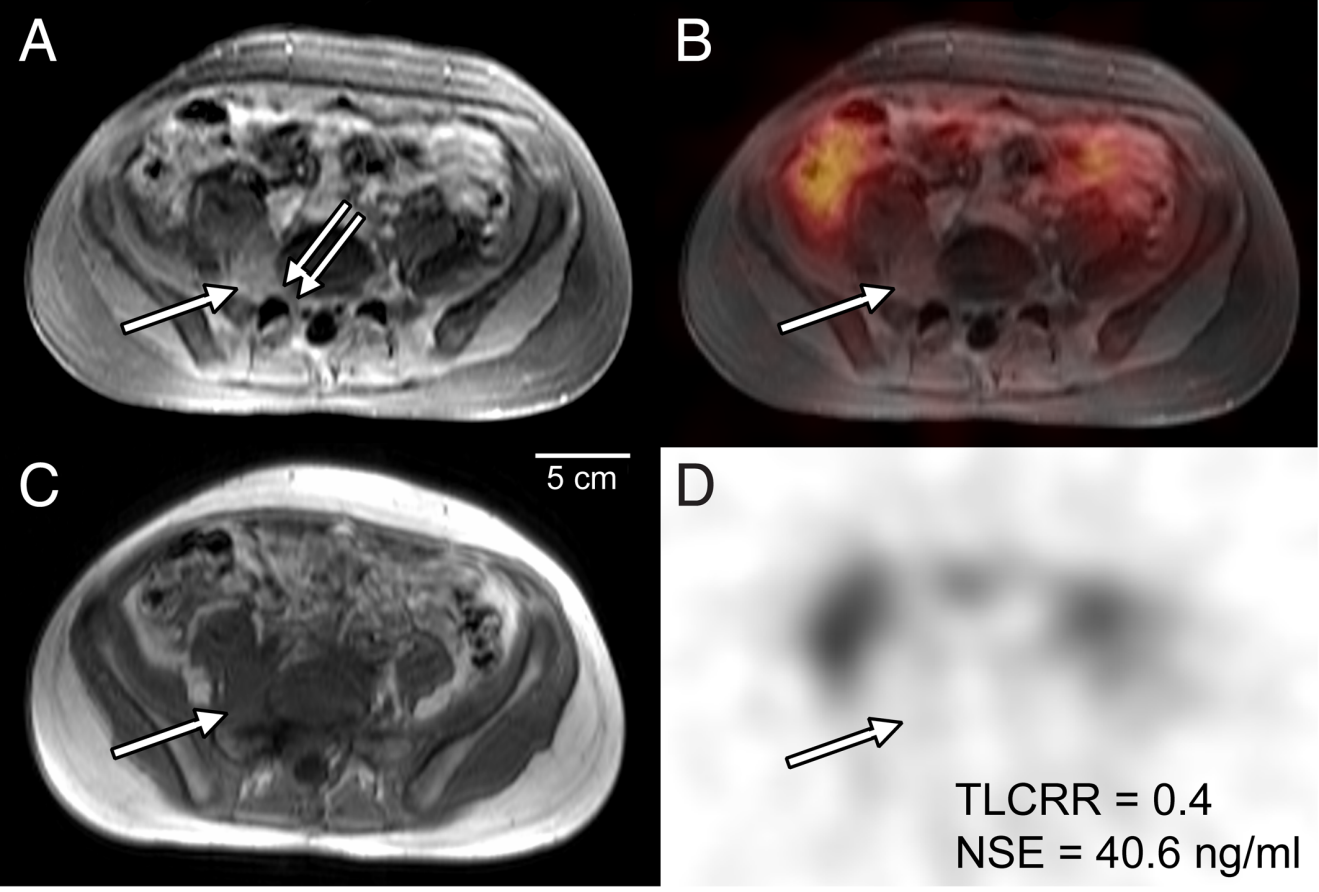
True positive prediction of unfavorable histopathology (UH) by combined TLCRR/NSE criteria in a 14 year old boy. T1-weighted axial MRI with **(A)** and without **(C)** contrast enhancement, ^123^I-MIBG SPECT **(D)** and fused SPECT/MRI **(B)** of the lower abdomen are shown. Tumor is depicted as contrast enhancing lesion in a paravertebral location **(A, arrow)** with infiltration of the L5 neuroforamen **(A, double arrow)**. Tumor-to-liver count-rate ratio (TLCRR) was < 2.0 **(D, false negative)**. Serum neuron-specific enolase (NSE) level was > 25.8 ng/ml **(D, true positive)**. Combined analysis resulted in a true positive finding for UH. Undifferentiated neuroblastoma was confirmed by histopathology following surgery.

**Fig 4:**
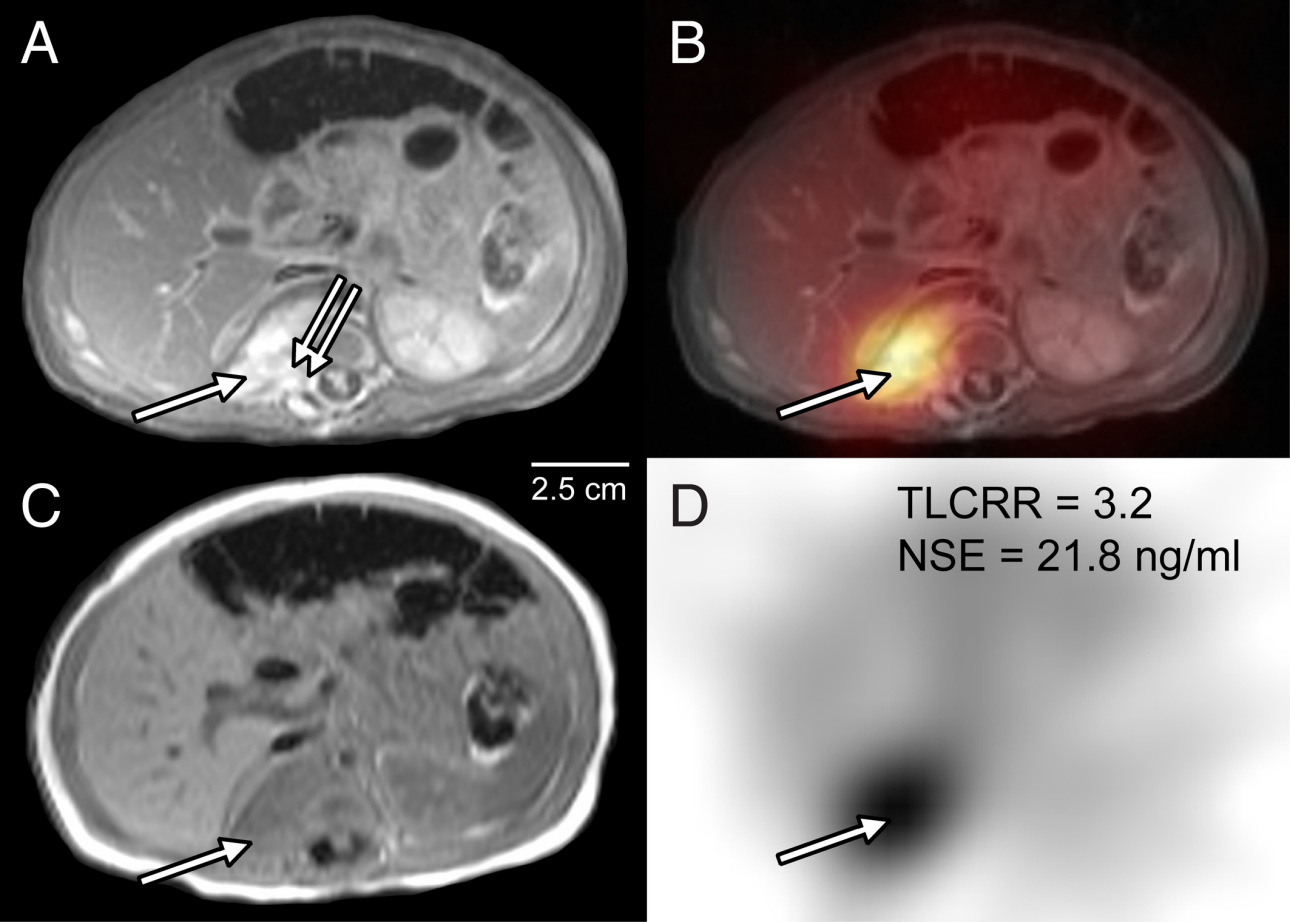
True positive prediction of unfavorable histopathology (UH) by combined TLCRR/NSE criteria in a 4 month old girl. T1-weighted axial MRI with **(A)** and without **(C)** contrast enhancement, ^123^I-MIBG SPECT **(D)** and fused SPECT/MRI **(B)** of the abdomen are shown. Images show a lesion with contrast enhancement **(A, arrow)** and infiltration of the L2 neuroforamen **(A, double arrow)**. Tumor-to-liver count-rate ratio (TLCRR) was > 2.0 **(D, true positive)**. Serum neuron-specific enolase (NSE) level was < 25.8 ng/ml **(D, false negative)**. Combined analysis resulted in a true positive finding for UH. Poorly differentiated neuroblastoma was confirmed by histopathology following surgery.

Additional analysis was performed by combining both parameters with stand-alone significant prediction according to ROC analysis: A positive result in either TLCRR or NSE was taken as a combined positive, whereas a negative result in TLCRR and NSE was taken as combined negative, given the high false negative rate for each single variable noted above. The diagnostic performance of TLCRR, NSE and their combined analysis for prediction of UH is given in Table 4. TLCRR and NSE were associated with equal relative risk of about two, but in the combined analysis there was a three-fold higher risk of UH in case of a combined positive result as compared to a combined negative result. Combined analysis reduced the false negative rate to five cases (as compared to 11/9 for stand-alone TLCRR/NSE), and furthermore gave the highest value for summed sensitivity plus specificity and accuracy scores. Figs 3 and 4 show two examples of true positive findings in the combined analysis.

**Table 4:** Performance of TLCRR, NSE and combined analysis for prediction of unfavorable histopathology. Sensitivity (SE), specificity (SP), positive predictive value (PPV), negative predictive value (NPV), and accuracy (AC) are each given in percent using optimal cut-off as determined by ROC analysis. Relative risk (RR) was calculated. TLCRR = tumor-to-liver count-rate ratio, NSE = neuron-specific enolase, ROC = receiver-operating-characteristic.

Parameter	Cut-off	SE	SP	PPV	NPV	AC	RR
TLCRR	2.0	68	100	100	54	77	2.2
NSE	25.8 ng/ml	74	85	93	55	77	2.1
Combined	-	85	85	94	69	85	3.0

## Discussion

MRI, ^123^I-MIBG scintigraphy and tumor marker analysis are widely used in the initial and follow-up assessment of patients with suspected neuroblastic tumor. All three modalities have established criteria for evaluation of tumor agressiveness and the extent of disease: These criteria include image-defined risk factors (IDRF) for the case of MRI, analysis of radiotracer uptake and distribution for the case of scintigraphy, and cut-off levels for urine or serum tumor markers. The aim of this study was to combine modalities so as to provide improved prediction of tumor histopathology as compared to the limited prediction from any single modality. We therefore tested ten variables for their accuracy to predict INPC unfavorable histopathology (UH), and combined the two individual parameters with good predictive value so as to obtain optimal diagnostic performance. Bonferroni correction was included to reduce the probability of false positive findings arsing from multiple testing.

The mean level of each tumor marker (MN, VMA, A, NA, HVA, DP, and NSE) was higher in patients with UH as compared to the FH group, however only serum NSE showed a significant correlation with histopathology in our analysis. Association between NSE level and INPC group has not been analyzed previously. However several trials have shown a significant correlation between serum NSE and patient prognosis. Lau et al. reported a high correlation between serum NSE and overall survival for all tumor stages in a cohort of 128 patients with neuroblastic tumor [17]. In their analysis, the risk for brief survival was increased by five-fold when the NSE level exceeded 100 ng/ml, whereas urine catecholamine levels did not generally correlate with survival [17]. Berthold et al. analyzed the prognostic value of different serum and urinary tumor markers for relapse or progression prediction in a series of 196 patients with neuroblastoma [18]. In that study, serum NSE proved to be the best predictor for local or distant progression, whereas urinary VMA and HVA both had low sensitivity [18]. We likewise found no significant correlation between urine catecholamine or catecholamine metabolite levels and high-risk histopathology. The urine metanephrine level did have some correlation with unfavorable histopathology (AUC-ROC 0.70), however was not considered significant after Bonferroni correction. Of course, our study is limited by its retrospective design and the low number of patients, which was a consequence of our stringent inclusion criteria. Therefore our multifactorial analysis did not have the statistical power necessary to detect a priori low correlation between histopathology and tumor marker concentrations; this might be best achieved in a prospective study with particular hypotheses based upon the present results.

The presence of infiltration or encasement of adjacent structures on MRI is considered a key risk factor for malignancy of neuroblastic tumors [12]. Indeed, we found that detection of ill-defined margins showed some correlation with UH, but this association did not reach statistical significance. Likewise, MRI size was not specifically associated with UH, as 11/13 (85%) of our patients with ganglioneuroma or intermixed ganglioneuroblastoma had lesions larger than five cm in diameter. However, MRI presents its own advantages for the diagnosis of neuroblastoma, given its high sensitivity for detecting bone marrow infiltration, and its precise delineation of the extent of intraspinal tumors [19]. Therefore, we assert that ^123^I-MIBG scintigraphy in conjunction with MRI give the optimal findings for risk stratification and surgery planning.

In a previous study Brans et al. categorized uptake of ^123^I-MIBG on planar images by using a seven-point visual scale in 26 patients with neuroblastic tumor [20]. In their analysis no correlation between ^123^I-MIBG uptake and histopathology was found, however a more accurate semiquantitative analysis of tracer uptake instead of visual rating was suggested. As compared to our previous study [6], we now implemented an improved version of our semiquantitative tumor-to-liver uptake ratio determined on SPECT images and found a close association between the intensity of ^123^I-MIBG uptake and histopathological subtype of neuroblastoma. This result also concurs with findings of Okuyama et al., who applied a four-grade visual scale for rating tracer uptake intensity in 23 neuroblastic tumor patients [21]. We now categorized our neuroblastic tumors into favorable (FH) and unfavorable (UH) to comply with INPC prognostic groups [1]. TLCRR had high specificity and a good AUC-ROC (0.86) based on the interpretation guideline presented by Swets et al. [22], but with quite low sensitivity due to the high rate of false negative findings for detection of UH (23%). The corresponding false negative rate of serum NSE was lower than for TLCRR (9 versus 11), and there was overlap for false negative results only in five patients. In our new combined analysis, a negative result had to have concordant negative findings for both TLCRR and serum NSE. By using this algorithm, the rate of false negative findings was reduced by approximately one half. In the combined analysis, the overall sensitivity, accuracy and relative risk were thus substantially improved as compared to each single parameter.

## Conclusion

Strong ^123^I-MIBG uptake and high levels of serum NSE are predictive of unfavorable histopathology in pediatric patients. Combined analysis of both parameters reduced false negative findings and improved accuracy for prediction of unfavorable histopathology in patients with neuroblastic tumor. If these findings should be confirmed in a larger group of patients, we argue that a combination of both parameters might perform well for pre-surgical and non-invasive prediction of patient risk. MRI parameters and urine catecholamine levels did not predict high-risk histopathology.
